# The Oncoprotein BRD4-NUT Generates Aberrant Histone Modification Patterns

**DOI:** 10.1371/journal.pone.0163820

**Published:** 2016-10-03

**Authors:** Barry M. Zee, Amy B. Dibona, Artyom A. Alekseyenko, Christopher A. French, Mitzi I. Kuroda

**Affiliations:** 1 Department of Genetics, Harvard Medical School, Boston, Massachusetts, United States of America; 2 Division of Genetics, Brigham and Women’s Hospital, Boston, Massachusetts, United States of America; 3 Department of Pathology, Brigham and Women’s Hospital, Boston, Massachusetts, United States of America; Texas A&M University, UNITED STATES

## Abstract

Defects in chromatin proteins frequently manifest in diseases. A striking case of a chromatin-centric disease is NUT-midline carcinoma (NMC), which is characterized by expression of NUT as a fusion partner most frequently with BRD4. ChIP-sequencing studies from NMC patients revealed that BRD4-NUT (B4N) covers large genomic regions and elevates transcription within these domains. To investigate how B4N modulates chromatin, we performed affinity purification of B4N when ectopically expressed in 293-TREx cells and quantified the associated histone posttranslational modifications (PTM) using proteomics. We observed significant enrichment of acetylation particularly on H3 K18 and of combinatorial patterns such as H3 K27 acetylation paired with K36 methylation. We postulate that B4N complexes override the preexisting histone code with new PTM patterns that reflect aberrant transcription and that epigenetically modulate the nucleosome environment toward the NMC state.

## Introduction

Histones assemble genomic DNA into chromatin and display many posttranslational modifications (PTMs) such as lysine acetylation. Nearly all PTMs are enzymatically regulated, for instance by lysine acetyltransferases (KATs) such as EP300. In addition PTMs can also be recognized by other non-histone proteins containing specific domains, for instance by bromodomain proteins such as BRD4. Many of these factors themselves regulate gene expression [1]. Consequently defects in the orchestration between enzymes and binding proteins can disrupt chromatin PTMs and the underlying transcriptional patterns, potentially manifesting in disease [2].

NUT-midline carcinomas (NMCs) are aggressive squamous cell cancers observed across a wide age distribution and are characterized by somatic expression of the *NUT* gene (also annotated as *NUTM1*), which is normally restricted to the germline [3]. Expression of the otherwise silent *NUT* gene arises from an in-frame fusion with an actively transcribed gene, most commonly with *BRD4* to yield BRD4-NUT (B4N). Remarkably B4N maps to approximately one hundred chromatin domains ranging from 100 to 2000 kilobases termed ‘megadomains’ that span genic and intergenic regions and that dwarf the 1 to 10 kilobase-sized domains occupied by most regulatory factors, for instance wild-type BRD4 [4].

How megadomains lead to the NMC pathology remains unclear. It has been hypothesized that in the process of creating these unusual structures B4N complexes ectopically regulate expression of key oncogenes, such as SOX2, MYC and TP63, though histone acetylation [5, 6]. ChIP-sequencing (ChIP-seq) has revealed that B4N co-localizes with histone H3 K27 acetylation (H3K27_ac_) in NMC cells, and additionally, when B4N is induced in non-NMC 293-TREx cells, the spreading of B4N coincides temporally and spatially with the spreading of H3K27ac [4]. The link between B4N and acetylation reflects both the bromodomain recognition of acetylated lysines and the NUT interaction with EP300 [6]. Indeed, when the NUT module alone is fused to a LacI binding domain and expressed in cells with a LacO-containing reporter, NUT alone is sufficient to concentrate high levels of H4 acetylation at the transgenic locus [7].

In contrast to antibodies directed against individual PTMs, liquid chromatography-mass spectrometry (LC-MS) can characterize a broader and more complex array of PTMs without complications such as epitope occlusion. Here we applied LC-MS based proteomics to characterize the histone PTMs that co-purify with B4N when ectopically expressed and crosslinked on chromatin in 293-TREx cells. We chose to perform our studies in these cells in order to simulate the expression of B4N in a negative megadomain background, similar to the presumptive initial NMC stages. As a positive control, we expressed wild type BRD4 (short form, 722 residues in length, hereafter abbreviated as BRD4_short_) which shares the same double bromodomains as B4N. In terms of amino acid sequence, BRD4-NUT is essentially BRD4_short_ fused to the full length NUT. As a negative control, we expressed L3MBTL3 which lacks bromodomains, maintains Polycomb group gene repression and is functionally unrelated to B4N. We crosslinked cells and performed affinity pulldowns to enrich for each bait-associated complex for proteomic analysis.

We expected to recover at minimum a similar level of histone acetylation from both the B4N and BRD4_short_ pulldowns. In fact we found that B4N uniquely enriches for hyper-acetylated and combinatorially modified histones distinct from BRD4_short_. Some of these combinations are comprised of PTMs that are rarely observed to co-localize or overlap together. We propose that the mechanisms of gene regulation by B4N are significantly different from BRD4_short_, namely that the B4N complex directly overrides the pre-existing histone PTM patterns, forming aberrant patterns that correspond to aberrant transcription linked to oncogenesis. The proteomic characterizations of PTMs found within B4N megadomains will serve as an invaluable resource for future NMC research.

## Materials and Methods

### Cloning/Tissue Culture

The 293-TREx-NBioTAP-BRD4NUT cell line was generated as described in our previous report [4]. The 293-TREx-NBioTAP-BRD4_short_ cell line was generated by FRT recombination with the plasmid pcDNA5 FRT/TO/N-BioTAP BRD4. To generate the pcDNA N-terminal BioTAP BRD4 plasmid itself, we used the Gateway recombination system to introduce the *BRD4* cDNA clone (CSBHuman Orfeome Collection; Internal ID: 71377) into pcDNA5 FRT/TO/NBioTAP vector. The 293-TRex-NBioTAP-L3MBTL3 cell line was generated by lentiviral infection with the pHAGE-NBioTAP L3MBTL3 plasmid. To generate the pHAGE N-terminal BioTAP L3MBTL3 plasmid itself, we used the Gateway recombination system to introduce the *L3MBTL3* cDNA clone (CSBHuman Orfeome Collection; Internal ID: 10868) into pHAGE NBioTAP vector. All cell lines were cultured in Dulbecco Modified Eagle Medium (Invitrogen) supplemented with 10% (v/v) fetal bovine serum (Atlanta Biologicals) and 1% (v/v) GlutaMAX (Invitrogen) and grown in a humidified incubator (Thermo Fisher) set at 37°C and 5% CO_2_.

### BioTAP-XL and LC-MS

Ten 15cm plates of 293-TREx cells containing expression cassettes for BioTAP-tagged BRD4_short_, L3MBTL3, and BRD4-NUT were induced by doxycycline at a concentration of 1 μg/ml overnight. Following induction, cells were processed to isolate the histones associated with the tagged baits as previously described [8]. Histones were derivatized with propionic anhydride both before and after trypsin digestion prior to desalting by in-lab constructed C_18_ STAGE-tips (3M). Desalted peptides were lyophilized by vacuum centrifuge and stored in -20°C. Peptides were re-suspended in HPLC buffer A (2.5% v/v acetonitrile, 0.1% formic acid, 97.4% water) and analyzed on an LTQ-Velos Orbitrap mass spectrometer (Thermo Fisher). Samples were autosampler loaded (FAMOS, LC Packings) onto C_18_ (reversed phase columns (Accucore 2.6 μm particle size, Thermo Fisher) heated to 60°C and electrosprayed into a Velos Orbitrap-Ion Trap (Thermo Fisher) mass spectrometer for analysis. MS data can be found at: https://dataverse.harvard.edu/dataset.xhtml?persistentId=doi:10.7910/DVN/6UMRIP.

In order to compare the relative levels of histone PTMs between the BioTAP-XL pulldown and genomic input, we first tested if both pulldown and input PTM data were normally distributed using the Shapiro-Wilk test. If data were normally distributed, we compared the change in PTM levels between pulldown and input using unpaired two-sample Welch’s t-test, which does not assume equal variance between the samples. All statistical tests were conducted using the respective built-in functions in the R software package (version 3.2.2, The R Foundation). Histone PTM levels are provided in S1 Table.

Total cellular histones were extracted as described in an earlier report [8]. Cells were lysed in Nuclear Extraction Buffer (20mM HEPES pH 7.6, 10% sucrose, 10mM NaCl, 3mM MgCl_2_, 0.2% Triton X-100, 1mM PMSF), followed by TEN400 buffer wash (10mM TrisCl pH 8.0, 1mM EDTA, 400mM NaCl, 1mM PMSF). Chromatin was extracted with TEN2000 buffer (10mM TrisCl pH 8.0, 1mM EDTA, 2M NaCl, 1mM PMSF), followed by acidification with sulfuric acid. The histones in the supernatant were precipitated with trichloracetic acid, washed with acetone, and re-suspended in 100mM ammonium bicarbonate (pH = 8.0).

For Western Blotting, the BioTAP-tagged baits were induced overnight with tetracycline. Cells were scraped and washed in Dulbecco’s Phosphate Buffered Saline (Boston BioProducts, 1mM PMSF) prior to lysis in RIPA buffer (140mM NaCl, 10mM TrisCl pH 8.0, 1mM EDTA, 1% Triton X-100, 0.1% SDS, 1mM PMSF) rotating for 15 minutes at 4°C. Supernatant was collected and protein concentration was approximated with Bradford reagent (Bio-Rad) using 595 nm absorbance. Approximately 60 micrograms of protein extract from each cell line were loaded per well on a TruPAGE 4–20% gradient gel (Sigma Aldrich). BioTAP-tagged baits were probed with peroxidase anti-peroxidase complex (1:1000 v:v, PAP, Sigma Aldrich, catalog number P1291) and loading control was determined with anti-GAPDH antibody (1:4000 v:v, Ambion, catalog number AM4300) and secondary HRP-conjugated anti-mouse antibody (1:4000 v:v, GE Healthcare, catalog number NA931). Chemiluminescence was detected with Clarity^™^ Western ECL Substrate (Bio-Rad) using an Odyssey FC Imager (Licor). Membrane was stained with Ponceau Red (Boston Bioproducts) to visualize total proteins.

### Imaging

293-TREx cells were grown on coverslips in a 6-well plate (Celltreat). HA- BRD4_short_ isoform or HA-B4N were induced by incubating cells overnight at 37°C in media containing 1 μg/mL tetracycline. Cells were fixed in 4% paraformaldehyde for 10 minutes at room temperature, then permeabilized in 0.3% Triton X-100 for 5 minutes at room temperature. Cells were then incubated in blocking buffer (PBS containing 0.3% Triton X-100 and 5% milk) for 30 minutes at room temperature. Cells were then incubated in primary antibody for 1 hour at room temperature. After washing in PBS, cells were incubated in secondary antibody for 1 hour at room temperature. Cells were washed in PBS and nuclei were counterstained with ProLong Gold anti-fade reagent with 4’,6-diamino-2-phenylindole (DAPI) (Life Technologies, catalog number P36935). Primary antibodies used were anti-HA.11 epitope tag (1:1000 v:v, BioLegend, catalog number 901513), anti-H3K18_ac_ (1:1000 v:v, Abcam, catalog number ab1191). Secondary antibodies included goat anti-mouse Alexa fluor 488 and goat anti-rabbit Alexa fluor 594 (1:1000 v:v, Life Technologies, catalog numbers A-11001 and A-11012). All antibodies were diluted in PBS containing 0.3% Triton X-100 and 5% milk. Images were acquired on an AxioSkop2 mot plus (Zeiss) fluorescence microscope and processed with Axiovision software (Zeiss Release 4.8).

Colocalization images were acquired by confocal microscopy. Confocal images were acquired on a Zeiss LSM 510 microscope using a 488nm, 543nm and multi-photon excitation at 780nm for the green, red and blue emitting fluorophores respectively; emission filters are band pass filters in the range of 500-530nm, 565-615nm, and 435-485nm. The objective is a Zeiss Plan-Apochromat 62x/1.4NA.

## Results

### Use of BioTAP-XL to identify chromatin factor associated histone PTMs

**Fig 1:**
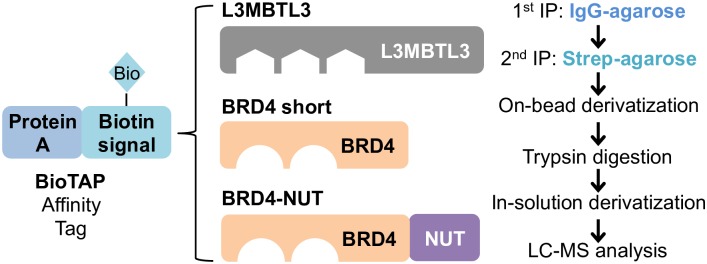
Experimental design to determine complex-specific histone PTMs. The BioTAP affinity tag, which combines both Protein A and a biotinylation acceptor sequence, was cloned into the N-terminus of the three different cDNAs. Tissue culture cells expressing the tagged baits were crosslinked with formaldehyde and sonicated prior to affinity pulldowns and on-bead derivatization.

We previously described the application of crosslinking and proteomics to analyze the histone PTMs associated with chromatin factors fused to the BioTAP affinity tag (Fig 1) [8]. While any tag could disrupt protein function, we demonstrated previously that BioTAP-B4N binds to the same genomic regions as endogenous un-tagged B4N in NMC patient cells by ChIP-seq [4]. Furthermore tagged B4N when expressed in 293-TREx cells forms a similar number of megadomains as seen in NMC cells, albeit in cell-type specific regions. As B4N also forms cell type-specific megadomains in different patients, this localization provides additional evidence that the BioTAP-tagged B4N functions similarly as the normally observed untagged oncoprotein, and that 293-TREx cells are a suitable system for studying the initial events in megadomain formation [4].

We generated 293-TREx cell lines expressing inducible BioTAP-L3MBTL3, BioTAP-BRD4_short_, and BioTAP-B4N (S1 Fig) [4]. After we induced bait expression, we crosslinked nuclei with formaldehyde and sonicated the chromatin suspension. We then performed affinity pulldowns of the tagged complex using IgG-agarose to bind the Protein A epitope in the first step, followed by streptavidin-agarose to bind the biotinylated tag in the second step. Finally we derivatized the streptavidin-bound complexes prior to trypsin digestion to release histone peptides of sufficient hydrophobicity and reduced charge state for reversed phase LC-MS.

To determine the enriched histone PTMs, we performed label-free relative quantification of the various modified peptides. We integrated the chromatographic peaks of each peptide and normalized the area against all modified forms of the same peptide backbone. This method of quantification differs from spectral counting, which measures the total number of MS/MS collected. If our baits did not enrich for specific histone PTM patterns, we would expect to recover essentially background levels of PTMs that are of a similar relative distribution as the genomic input. For certain peptides such as the mono-acetylated H3 18–26 peptide (K_18_QLATK_23_AAR), where the single acetyl group can localize to either K18 or K23 and where these two isoforms can co-elute, we additionally relied on MS/MS quantification. We examined the average MS/MS across the chromatographic peak and determined the relative fragment ion ratios characteristic of either isoform.

Our experimental variability can be approximated by how consistently we recover peptides expected to be equally present across our samples. Most human canonical H2A histones, such as type 1-B/E and 2-A, contain either a threonine or serine at position 16. Assuming this substitution is indiscernible by our baits, we should recover peptides spanning the T16/S16 position (AKAKT_16_R and AKAKS_16_R) at a consistent frequency. We observed that the fold difference in the T16/S16 peptide levels did not differ by more than 20% of the sample mean value (S2 Fig). When interpreting our data, fold changes greater than double this threshold (that is, 0.4 times) are unlikely due to expected technical variability.

We next tested whether the expression of B4N might alter global histone PTM levels that consequently would skew the results, similar to the effects of adding a pan-deacetylase inhibitor. We extracted total cellular histones from 293-TREx cells before and after B4N induction and quantified the PTM levels. Aside from a decrease in H3K79 di-methylation (me2) and tri-methylation (me3) upon induction, most PTMs remain unchanged (S3 Fig). Thus, PTMs enriched by B4N at greater than 0.4× fold of the control pulldowns are unlikely to be due to global effects.

### BRD4-NUT bound histones have overall distinct PTMs from BRD4_short_ bound histones

To understand how different the overall histone PTM patterns recovered from the B4N pulldown are with respect to the other samples, we determined the Pearson correlation coefficient r for pairwise comparisons between the samples. We find that histone PTMs of replicates are linearly correlated with one another, while B4N histone PTM patterns are significantly non-correlated with genomic or BRD4_short_ PTM patterns (S4 Fig). Thus the B4N-associated histone PTM patterns when considered collectively are distinct from BRD4_short_ or input genomic PTM patterns.

### BRD4-NUT complex drives H3K18 toward the acetylated state

**Fig 2:**
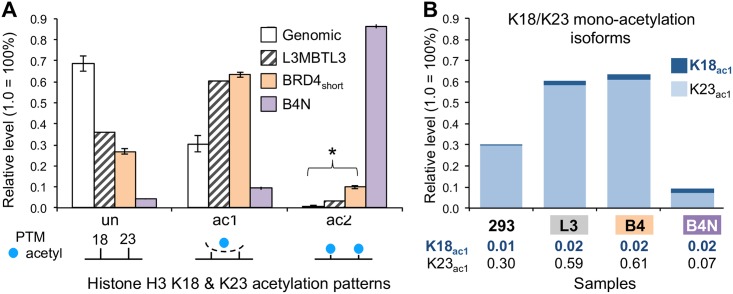
Quantification of H3 K18 and K23 acetylation enrichment. (A) Relative levels of unmodified, mono-acetylation, and di-acetylation of the H3 peptide spanning K18 and K23 in genomic and immunoprecipitated histones. Note that the mono-acetylation values combine K18_ac1_K23_un_ and K18_un_K23_ac1_ together. Error bars of genomic and BRD4_short_ samples represent standard deviation of 4 replicates. Error bars of B4N sample represent range of 2 replicates. Asterisk denotes significant difference in ac2 levels between input and BRD4_short_ (p < 0.05). (B) Resolving the mono-acetylation patterns in Fig 2A into the two isoforms for each sample. Relative levels are provided below each bar corresponding to K18ac1 (top bold) and K23ac1 (bottom) and are the same scale as in panel A. For instance, the 30% H3K23ac1 in the genomic sample is relative to unmodified, H3K18ac1, and H3K18K23ac2 as seen in Panel A. Note that most of the mono-acetylated in the samples is K23ac1. 293 refers to genomic histones, L3 refers to L3MBTL3 associated histones, and B4 refers to BRD4_short_ associated histones.

To identify specific PTMs enriched by our baits, we first analyzed the H3 18–26 peptide spanning K18 and K23. EP300 and CREBBP are the main enzymes that acetylate K18 and K23 [9]. We found that pulldown of BRD4_short_ significantly enriched for di-acetylated H3K18_ac_K23_ac_ relative to input (Welch: t = -21.19, df = 3.7172, p = 5.1×10^−5^; Shapiro-Wilk: genomic p = 0.44, BRD4_short_ genomic p = 0.95). Although B4N did not enrich for mono-acetylation relative to the other baits, B4N did enriched for di-acetylation more than 8-fold over BRD4_short_ (Fig 2A). To determine whether a particular mono-acetyl isoform was depleted, we resolved the mono-acetylated peak into K18_ac1_ and K23_ac1_ (Fig 2B). Genomic levels of K23 mono-acetylation generally exceed K18 mono-acetylation in human tissue culture lines [10]. The K23_ac_ majority is reflected in the BRD4 and L3MBTL3 pulldowns. Our data suggest that B4N initially recognizes histones mono-acetylated at K23 in a potentially similar manner as wild-type BRD4. The B4N complex, and more specifically the EP300/CREBBP binding partner, would acetylate the next available unmodified residue at K18 and convert the mono-acetylated K18_un_K23_ac_ state into the di-acetylated K18_ac_K23_ac_ state. We do not expect a similar enrichment for di-acetylation with wild type BRD4, which exhibits no significant interaction with EP300.

**Fig 3:**
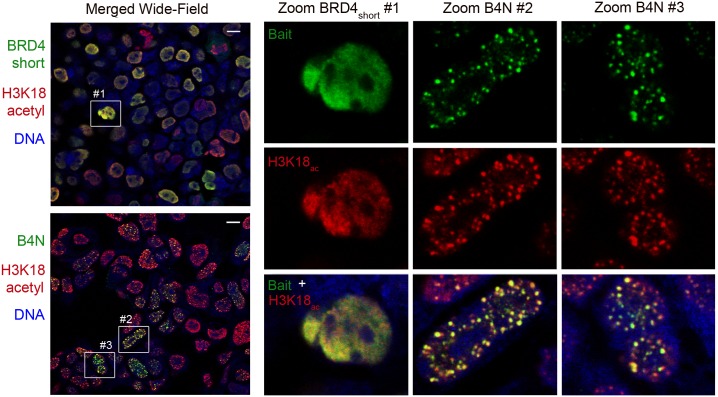
Formation of nuclear foci enriched in H3K18 acetylation by B4N and not BRD4_short_. Confocal microscopy of 293-TREx cells with inducible HA-B4N and HA-BRD4_short_ stained with anti-HA (green), which labels the bait protein, and anti-H3K18ac (red). Scale bar represents 10 micrometers. Zoomed-in images are provided to illustrate difference between H3K18_ac_ granules in BRD4_short_ cells and larger H3K18_ac_ foci in B4N cells. See S5 Fig for non-confocal imaging and S6 Fig for separate green and red confocal layers of the same wide-field image of B4N-expressing cells.

The proteomic data suggest that B4N enriches for H3 K18 acetylation by driving local chromatin towards a hyper-acetylated state. As this was not a modification that we assayed in our previous candidate approach, we induced BRD4_short_ and B4N with a FLAG-HA tag in 293-TREx lines and assayed the localization of H3K18_ac_. If B4N-dependent PTM conversion were occurring, we expected to detect a significant change in K18 acetylation patterns upon B4N expression but not BRD4 expression. We found that in the non-induced state, endogenous H3K18_ac_ is distributed throughout the nucleus (S5 Fig). When wild type BRD4_short_ is induced, H3K18_ac_ remains diffuse throughout the nucleus with low levels of granularity (Fig 3, S5 Fig). In contrast, when B4N is induced, H3K18_ac_ forms large foci that resemble NMC megadomains not seen when BRD4_short_ is expressed (Fig 3, S5 and S6 Figs). We observed co-localization between the large H3K18_ac_ foci and B4N (Fig 3). Thus the proteomic and microscopy experiments support a model where the B4N oncoprotein dramatically transforms histone acetylation patterns within nuclear foci that were previously revealed to comprise the NMC megadomains [4].

### BRD4-NUT complex associates with hyper-acetylated H4

Like histone H3, histone H4 can also be extensively acetylated. Aside from N-terminal acetylation, H4 can be mono-, di-, tri-, and tetra-acetylated at K5, K8, K12, and K16. H4 acetylation is broadly correlated with active transcription in a variety of organisms, with the notable exception that H4K12_ac_ localizes over heterochromatin in fruit flies and budding yeast [11]. Interestingly acetylation at these distinct residues have distinct metagene distributions. For instance in mouse ES cells, H4K16_ac_ is strongly enriched within a few kilobases both upstream and downstream of transcriptional start sites while H4K8_ac_ is more modestly enriched. H4K5_ac_ is relatively depleted at these sites [12]. To further illustrate the differences between the H4 acetylation residues, H4K5_ac_ and H4K8_ac_ are both enriched in non-small cell lung cancers that include squamous cell carcinomas and adenocarcinomas compared to normal lung parenchyma, while H4K12_ac_ and H4K16_ac_ are depleted in the same cancers [13].

**Fig 4:**
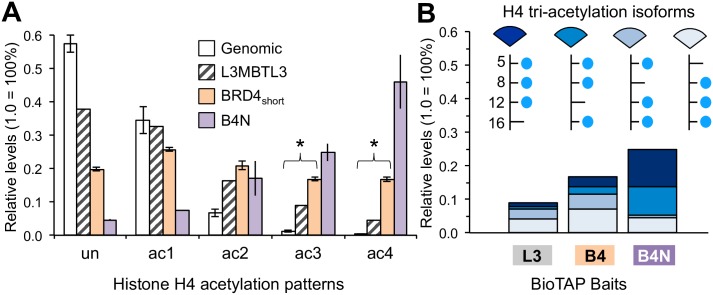
Quantification of H4 K5, K8, K12, and K16 acetylation enrichment. (A) Relative levels of unmodified, mono-acetylation, di-acetylation, tri-acetylation, and tetra-acetylation of the H4 peptide spanning K5, K8, K12, and K16 in genomic and immunoprecipitated histones. Error bars of genomic and BRD4_short_ samples represent standard deviation of 4 replicates. Error bar of B4N sample represents range of 2 replicates. Asterisks denote significant difference in ac3 and ac4 levels between input and BRD4_short_ (p < 0.05). (B) Resolving the tri-acetylation patterns in Fig 4A into the four isoforms for each sample. The two most abundant tri-acetylated isoforms in the B4N samples are K5K8K12ac3 and K5K8K16ac3. Abbreviations are the same as in Fig 2B.

We quantified the acetyl states of the H4 4–17 peptide (GK_5_GGK_8_GLGK_12_GGAK_16_R) that spans K5, K8, K12, and K16 from the pulldowns. BRD4_short_ significantly enriched for H4 tri-acetylation (Welch: t = -46.967, df = 5.1112, p = 6.2×10^−8^; Shapiro-Wilk: genomic p = 0.14, BRD4_short_ p = 0.78) and tetra-acetylation (Welch: t = -48.88, df = 3.0628, p = 1.5×10^−5^; Shapiro-Wilk: genomic p = 0.19, BRD4_short_ p = 0.87) relative to input (Fig 4A). Strikingly B4N enriched for both H4 tri- and tetra-acetylation to an even greater extent than BRD4_short_ relative to input, such that over half of the histone H4 recovered by B4N were either tri- or tetra-acetylated (Fig 4A). To interrogate whether B4N was preferentially associating with specific acetylated residues, we resolved the H4 tri-acetylated data into the four individual isoforms: K5/K8/K12_ac3_, K5/K8/K16_ac3_, K5/K12/K16_ac3_, and K8/K12/K16_ac3_ (Fig 4B). We observed far greater enrichment of the K5/K8/K12_ac3_ and the K5/K8/K16_ac3_ isoforms by B4N than either BRD4_short_ or L3MBTL3.

A common feature of both isoforms is acetylation at K5 and K8. Since the double bromodomains of B4N are identical to wild type BRD4_short_, we predict that the intrinsic acetyl lysine specificity of B4N is similar to wild type BRD4_short_. Other groups have performed in vitro peptide binding assays showing that the individual bromodomains of BRD4 bind preferentially to K5 acetylation [14]. The same assays also revealed that the EP300 bromodomain recognizes K5_ac_, K8_ac_, and K12_ac_, but not K16_ac_. Consistent with these in vitro findings, in vivo imaging experiments from other groups revealed significant co-localization between K5_ac_ and K12_ac_ with CREBBP and between K8_ac_ with EP300. Little co-localization was observed between K16_ac_ with either KAT [15]. Thus the enrichment for H4 isoforms containing K5 and K8 acetylation as measured by our experiments may reflect the selectivity of B4N interactors as well as B4N itself.

### BRD4-NUT complex enriches for K27 acetylation and K36 methylation in the H3.3 variant

We demonstrated that B4N significantly enriches for H3K18 and K23 acetylation, as well as H4K5 and K8 acetylation relative to control pulldowns in 293-TREx cells. We also examined acetylation on the H3 9–17 peptide (K_9_STGGK_14_APR) spanning K9 and K14. We found that BRD4_short_ significantly enriched for di-acetylated H3K9_ac_K14_ac_ over input (Welch: t = -13.162, df = 3.0392, p = 8.9×10^−4^; Shapiro-Wilk: genomic p = 0.10, BRD4_short_ p = 0.72). Additionally we found that B4N enriches for di-acetylation at both H3 K9 and K14 by 2-fold over BRD4_short_ (S7 Fig). These results support a model where B4N recognizes and generates histone hyper-acetylation at large genomic regions.

We next wanted to study how B4N might alter methylation by investigating the methylations on the H3 73–83 peptide (EIAQDFK_79_TDLR) spanning K79. We found that BRD4_short_ significantly enriched for H3K79 mono-methylation over input (Welch: t = -4.5686, df = 2.6545, p = 0.02552; Shapiro-Wilk: genomic p = 0.15, BRD4_short_ p = 0.53). We found that B4N enriched for K79_me1_, but not K79_me2_ (S8 Fig), to a greater extent over BRD4_short_. The lack of K79_me2_ enrichment may be skewed due to the decrease in global K79_me2_ levels upon B4N induction (S3 Fig). Other groups have found that H3K79_me1_ is highly correlated with actively expressed genes containing low CpG content promoters [16]. While BRD4 and the H3K79 methyltransferase DOT1L do not physically interact, studies in mixed lineage leukemia have suggested DOT1L methylates and establishes a chromatin state that is permissive for acetylation and BRD4 recruitment [17]. With respect to our results, genes already decorated with H3K79_me1_ may be primed for EP300 acetylation and subsequent B4N recruitment.

Following the K79 data, we investigated the methyl and acetyl marks enriched by B4N by focusing on the variant H3.3 27–40 peptide (K_27_SAPS_31_TGGVK_36_KPHR) that spans K27 and K36. The H3 data described so far in this paper could not distinguish between canonical H3 or variant H3.3, as the two proteins yield identical peptides at the K18/K23 and K9/K14 positions. However the alanine and serine substitution at position 31 allows us to use LC-MS to distinguish peptides spanning K27 and K36 from the canonical and variant proteins. In fact K27 and K36 PTM-specific antibodies likely cross-react with both canonical and variant H3 proteins.

In contrast to canonical H3, H3.3 is expressed independently of DNA replication and is selectively incorporated into active genes by the HIRA histone chaperone [18]. For both canonical and variant H3, di- and tri-methylation at K27 is correlated with gene silencing, while mono-methylation at K27 is correlated with active expression [19]. Di- and tri-methylation of K36 is correlated with gene activation and localized to genic regions [20]. Though distinct enzymes are responsible for modifying these residues, studies in mouse embryonic stem cells suggested that there is cross-regulation between the enzymes, where knockdown of the K36 methyltransferase Setd2 leads to a reduction in K27 mono-methylation [19].

**Fig 5:**
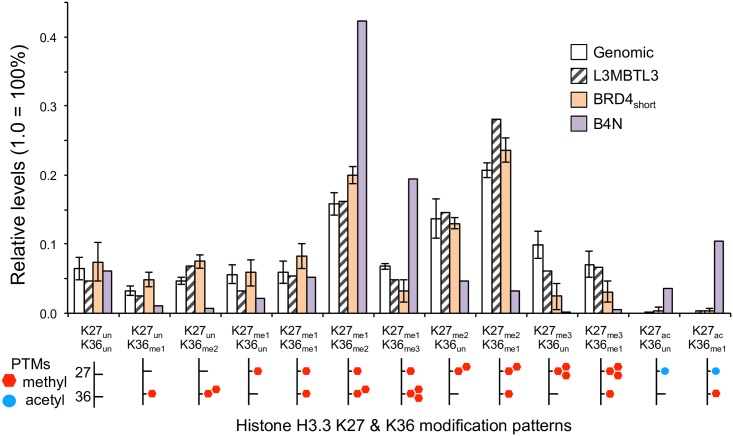
Quantification of variant H3.3 K27 and K36 modification enrichment. Relative levels of combinatorially modified forms of K27 and K36 methylation and acetylation. Error bar of genomic and BRD4_short_ sample represents standard deviation of 4 replicates. Note the significant enrichment for K27 mono-methylation paired with K36 di- and tri-methylation by B4N, as well as K27 acetylation paired with K36 mono-methylation.

Our proteomic experiments revealed that B4N does not enrich for H3.3 containing only K27 methylation or only K36 methylation (Fig 5). This trend is especially pronounced for di-methylated K27 (K27_me2_K36_un_) and tri-methylated K27 (K27_me3_K36_un_), where B4N strongly depletes for both methyl forms. Rather than enriching for individual methyl PTMs, we found that B4N enriches for combinatorial PTMs such as mono-methylated K27 paired with di-methylated K36 (K27_me1_K36_me2_) as well as with tri-methylated K36 (K27_me1_K36_me3_). Notably K36 tri-methylation is rarely detected without the simultaneous occurrence of K27 mono-methylation in human cells.

Consistent with previous genomics data [4], our current proteomics data revealed that B4N enriches for H3.3 containing acetylated K27 paired with unmodified K36 (K27_ac_K36_un_) and paired with mono-methylated K36 (K27_ac_K36_me1_). The K27_ac_K36_me1_ combination is especially intriguing as this peptide is often below the quantifiable limit in other samples. Additionally K36 mono-methylation and K27 acetylation generally have distinct metagene profiles, with K36_me1_ in genic regions and K27_ac_ in enhancers [21]. MS/MS confirmed that the acetyl group is on K27 and the methyl group (with the propone group from the derivatization) on K36 (S9 Fig).

We observed similar trends of K27/K36 enrichment with canonical H3, albeit with more subtle enrichment for K27_ac_ (S10 and S11 Figs). Since BRD4_short_ does not significantly enrich for K27_me1_K36_me2_, K27_me1_K36_me3_, or K27_ac_K36_me1_ to the same extent as B4N, the enrichment of these modified forms by B4N is likely attributed to the NUT module. Given the selective interaction between B4N and EP300/CREBBP, it is possible that K27_ac_K36_me1_ arises from the B4N complex acetylating genomic regions already mono-methylated at K36.

It is more difficult though to interpret the B4N enrichment of K27_me1_K36_me2_ and K27_me1_K36_me3_. For instance the formation of K27_me1_K36_me3_ could signify mono-methylation at K27 of a pre-existing K36_me3_ protein or, reciprocally, tri-methylation at K36 of a pre-existing K27_me1_ protein. Both the short form of BRD4 and the B4N oncoprotein retain the extra-terminal domain that interacts with the H3K36 methyltransferase WHSC1L1 (also annotated as NSD3) [22]. Hence, the enrichment of K36 methylation is unlikely to be accounted by selective interaction with WHSC1L1.

**Fig 6:**
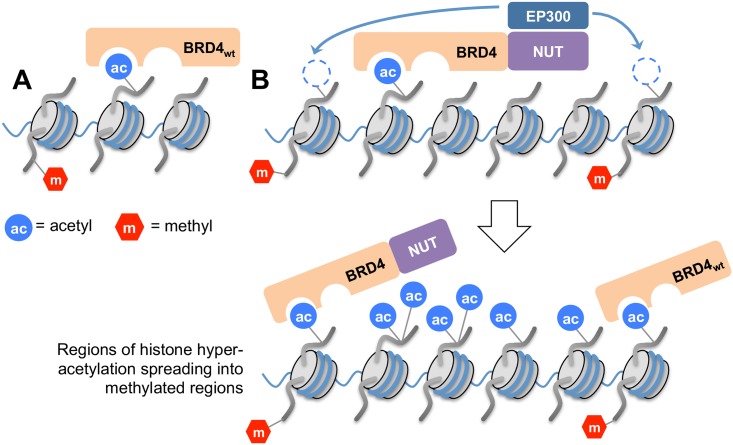
Model for ectopic histone PTM activation by the B4N complex. (A) Wild type BRD4 recognizes regions that are already acetylated. (B) B4N complex binds to regions already acetylated. These regions may also be already marked with H3K79 mono-methylation. With the EP300 binding partner, the B4N complex targets adjacent chromatin for additional acetylation, for instance at H3K18. This creates additional binding sites for B4N, allowing B4N to spread farther than wild type BRD4 and potentially into regions containing H3K36 methylation. Wild type BRD4 can also co-localize with B4N as megadomains are established.

It is possible that the apparent enrichment for K36 methylation paired with H3K27 mono-methylation represents a secondary aspect of B4N spreading. In particular, B4N appears to form megadomains primarily through the binding of pre-existing histone acetylation that may be devoid of H3K36 methylation, for instance in regulatory non-coding enhancer regions that instead contain H3K27 acetylation. This is supported by previous ChIP-seq studies of B4N induction in 293-TREx cells that revealed B4N initially binds to enhancers before forming megadomains [4]. As such, we propose the following model for B4N with respect to BRD4_short_ (Fig 6). BRD4_short_ binds to acetylated chromatin via its double bromodomains. B4N, similar to BRD4_short_, would initially bind to non-genic regions that contain pre-existing acetylation. After initial binding though, B4N complexes, and in particular its EP300 binding partner, may generate new acetylations at H3K18 in adjacent chromatin, for instance into genic regions that do contain pre-existing H3K36 tri-methylation. This spreading could account for the high enrichment of H3K18 acetylation as well as apparent enrichment of K27_me1_K36_me3_ by B4N. Furthermore, the initial binding and propagation from active enhancers by B4N complexes could account for the higher enrichment of H3.3 K27_ac_K36_un_ and K27_ac_K36_me1_ relative to canonical H3.1/H3.2, since H3.3-containing nucleosomes are selectively found in these regulatory regions.

## Discussion

The histone code describes how PTMs are linked to gene regulation through the relevant non-histone factors that form, remove, and recognize PTMs. From our proteomic studies into how BRD4-NUT binds to chromatin, we uncovered complex histone PTM patterns associated with the fusion oncoprotein. We believe these combinatorial PTMs arise from the activities of B4N and its associated enzymes such as EP300. Our data suggest that the B4N complex overrides the pre-existing histone code and forms an epigenetic state corresponding to aberrant transcription.

Previous sequencing experiments have shown that B4N domains are generally delimited within topologically associated domains (TADs) [4]. It remains possible that the PTM patterns we observe are due to B4N complexes aggregating pre-existing PTMs within a given TAD without any de novo PTM conversion. However our previous experiments showed that when B4N was induced in 293-TREx cells, H3K27ac spreads from localized pre-existing peaks into much broader regions that tracked with the emergence of B4N megadomains [4]. The increase in H3K27ac over genomic regions previously devoid of acetylation suggests new modification formation in response to B4N.

A more direct future experiment to determine whether de novo PTM formation and turnover is occurring would be to induce B4N and BRD4_short_ with pulse labeling of isotopically heavy glucose, which labels new acetyl groups. If our model for PTM generation within B4N domains were correct, we expect the acetyl labeling efficiency in B4N-associated chromatin relative to non-associated chromatin to be higher than BRD4_short_-associated chromatin relative to non-associated chromatin.

With respect to the dynamics of histone PTM regulation, it is interesting to note that the rate of histone H4 acetylation turnover in human foreskin fibroblasts is nearly double when cells are actively proliferating as opposed to when cells are quiescent [23]. As NMC cells resume proliferation due to oncogene activation, the efficiency of acetylation may accelerate and further facilitate the formation of megadomains. Thus in the treatment of NMCs, it would be advantageous to block the cycle of aberrant PTM formation either by directly knocking out B4N itself or using small molecules that selectively inhibit the B4N-dependent interaction with EP300/CREBBP. Such approaches should minimize off-target effects against wild type BRD4 and could synergistically act with other compounds such as Flavopirodol, which has shown promising results in inducing cytotoxicity in NMC cells compared to non-NMC controls [24].

One intriguing aspect of NMC is the sequestration of B4N-bound enzymes. Previous experiments have shown that B4N has lower intra-nuclear mobility than wild type BRD4, presumably reflecting the B4N complex being trapped within regions of concentrated histone acetylation [25]. The titration of B4N components within megadomains could lead to an imbalance of enzymatic regulation outside of megadomains, potentially resulting in hypo-acetylation. Indeed treatments that administer the lysine deacetylase inhibitor Vorinostat and that presumably restore the histone acetylation and transcriptional patterns of these non-megadomain regions have shown promise in treating NMC patients [26]. To clarify the mechanisms of KAT sequestration, future studies should characterize the histone PTMs in regions outside of megadomains before and after therapeutic intervention. Though we focused solely on BRD4-NUT in this report, future research should also determine whether other NMC fusion proteins, such as BRD3-NUT, NSD3-NUT, and even other BRD4-NUT proteins with different fusion junctions, enrich for similar modifications [25, 27–29]. Finally, it would be critical to study resected NMC patient tissues and perform affinity pulldowns to identify NUT-fusion protein associated modifications. We estimate based on nascent RNA-sequencing that the level of B4N induced in non-NMC cells is approximately 5-fold higher than endogenous B4N expressed in NMC cells [4]. Studying the endogenous NUT-fusion protein in NMC tissues would avoid bait over-expression.

In summary, B4N enriches for chromatin containing multiple acetyl and methyl groups. It would have been difficult to assay such complex isoforms with conventional antibody-based approaches. Furthermore it would have been challenging to isolate B4N-bound complexes with conventional native affinity approaches. The use of formaldehyde and sonication effectively preserves and solubilizes complexes containing DNA, RNA, and protein that could otherwise be destabilized with MNase digestion and salt extractions. In this respect, BioTAP-XL will be an invaluable method to understand how aberrant regulation of the histone code may drive other chromatin-centric diseases.

## Supporting Information

S1 Fig Western Blot of induction of BioTAP-tagged baits in 293-TREx cells. Western Blot analysis of protein extracts from tetracycline induced 293-TREx that are non-transfected (Lane 1), expressing BioTAP-L3MBTL3 (Lane 2; 100kd expected molecular weight), BioTAP-BRD4short (Lane 2; 100kd expected molecular weight) (Lane 3), and BioTAP-B4N (Lane 4; 220 kd expected molecular weight). PAP detects BioTAP-tagged baits. GAPDH and Ponceau staining reveal equal loading of extracts.
(TIF)

S2 Fig Consistency of BioTAP-XL experiments and global non-specific effects of B4N induction. Recovery of H2A variant peptides across genomic and immunoprecipitated chromatin spanning endogenous substitutions at position 16 (serine compared to threonine). Error bar of genomic sample and BRD4_short_ represents standard deviation of 4 replicates while error bar of B4N sample represents range of 2 replicates.
(TIF)

S3 Fig Test for global non-specific effects of B4N induction. Comparison of global histone PTM levels in 293-TREx cells before and 7 hours after B4N induction. Each point in the plot represents a PTM. Relative PTM levels were determined as described in the report, where the area under the peak for each modified peptide was normalized to the sum of all the areas of the unmodified and modified forms of a given peptide backbone. The relative quantification was performed for both 0hr and 7hr induction, and the values per PTM at the two timepoints were log_2_ transformed and multiplied by -1. Note that, aside from H3K79 di- and tri-methylation, most PTMs on a genome wide scale do not significantly change upon B4N induction.
(TIF)

S4 Fig Linear correlation between histone PTM patterns. Pearson’s coefficient r was determined for the collective relative abundances of histone PTMs in pairwise comparisons between our samples (genomic input, BRD4_short_, and B4N). Above the diagonal are scatterplots of each comparison, with each point representing a histone PTM. For each comparison, we tested the likelihood that we would have observed the sample correlation if our null hypothesis of r being 0 were true. Thus low p-values suggest a high likelihood that there is a correlation. Statistical values are shown below the diagonal, where p-values are bolded and the lower and upper 95% confidence interval for Pearson’s coefficient are shown below. We find that within replicates, our MS-based PTM quants are tightly correlated. However, the B4N samples are not correlated (positively or negatively) with either input or BRD4_short_, suggesting that the B4N-associated PTM patterns as a whole are highly distinct from input and BRD4_short_.
(TIF)

S5 Fig Non-confocal imaging of BRD4_short_ and B4N with H3K18ac. Images of 293-TREx cells expressing inducible HA-B4N and HA-BRD4_short_ stained with anti-HA (green), which labels the bait protein, and anti-H3K18ac (red), acquired on non-confocal widefield fluorescence microscopy. Scale bar represents 10 micrometers.
(TIF)

S6 Fig Separate layers of confocal imaging of B4N and H3K18ac. Same confocal image as Fig 3 with separate panels for the green (left, stained for BRD4-NUT bait) and red (right, stained for H3K18ac) channels for clarity.
(TIF)

S7 Fig Quantification of H3 K9, K14 acetylation enrichment. Relative levels of unmodified, mono-acetylation and di-acetylation of the H3 peptide spanning K9 and K14 in genomic and immunoprecipitated histones. Error bar of genomic and BRD4_short_ sample represents standard deviation of 4 replicates. Error bar of B4N sample represents range of 2 replicates. Asterisk denote significant difference in H3K9K14ac2 levels between input and BRD4_short_ (p < 0.05).
(TIF)

S8 Fig Quantification of H3 K79 methylation enrichment. Relative levels of unmodified, mono-methylated and di-methylated H3K79 in genomic and immunoprecipitated histones. Error bar of genomic and BRD4_short_ sample represents standard deviation of average of 3 replicates. Error bar of B4N sample represents range of 2 replicates. Asterisk denote significant difference in K79me1 levels between input and BRD4_short_ (p < 0.05).
(TIF)

S9 Fig MS/MS of variant H3.3 K27acK36me. Annotated tandem mass spectrum of the H3.3 peptide containing K27 acetylation and K36 monomethylation. Numbers above and below peptide sequence correspond to nominal masses of b and y fragment ions. Bolded masses indicate those ions annotated from the spectrum. Pr = propone group from derivatization.
(TIF)

S10 Fig Quantification of canonical H3.1/H3.2 K27 and K36 PTM levels. Relative levels of combinatorially modified forms of K27 and K36 methylation and acetylation. Error bar of genomic and BRD4_short_ sample represents standard deviation of 4 replicates. Note the significant enrichment for K27 mono-methylation paired with K36 di- and tri-methylation by B4N.
(TIF)

S11 Fig MS/MS of canonical H3.1/H3.2 K27acK36me. Annotated tandem mass spectrum of the canonical histone H3 peptide containing K27 acetylation and K36 monomethylation. Numbers above and below peptide sequence correspond to nominal masses of b and y fragment ions. Bolded masses indicate those ions annotated from the spectrum. Pr = propone group from derivatization.
(TIF)

S1 Table Relative quantification of histone PTM levels. (XLSX)
