# Supplementary figures and images for: The Oncoprotein BRD4-NUT Generates Aberrant Histone Modification Patterns
Source: PLoS One. 2016 Oct 3;11(10):e0163820. doi: 10.1371/journal.pone.0163820 (PMC5047744; doi:10.1371/journal.pone.0163820)

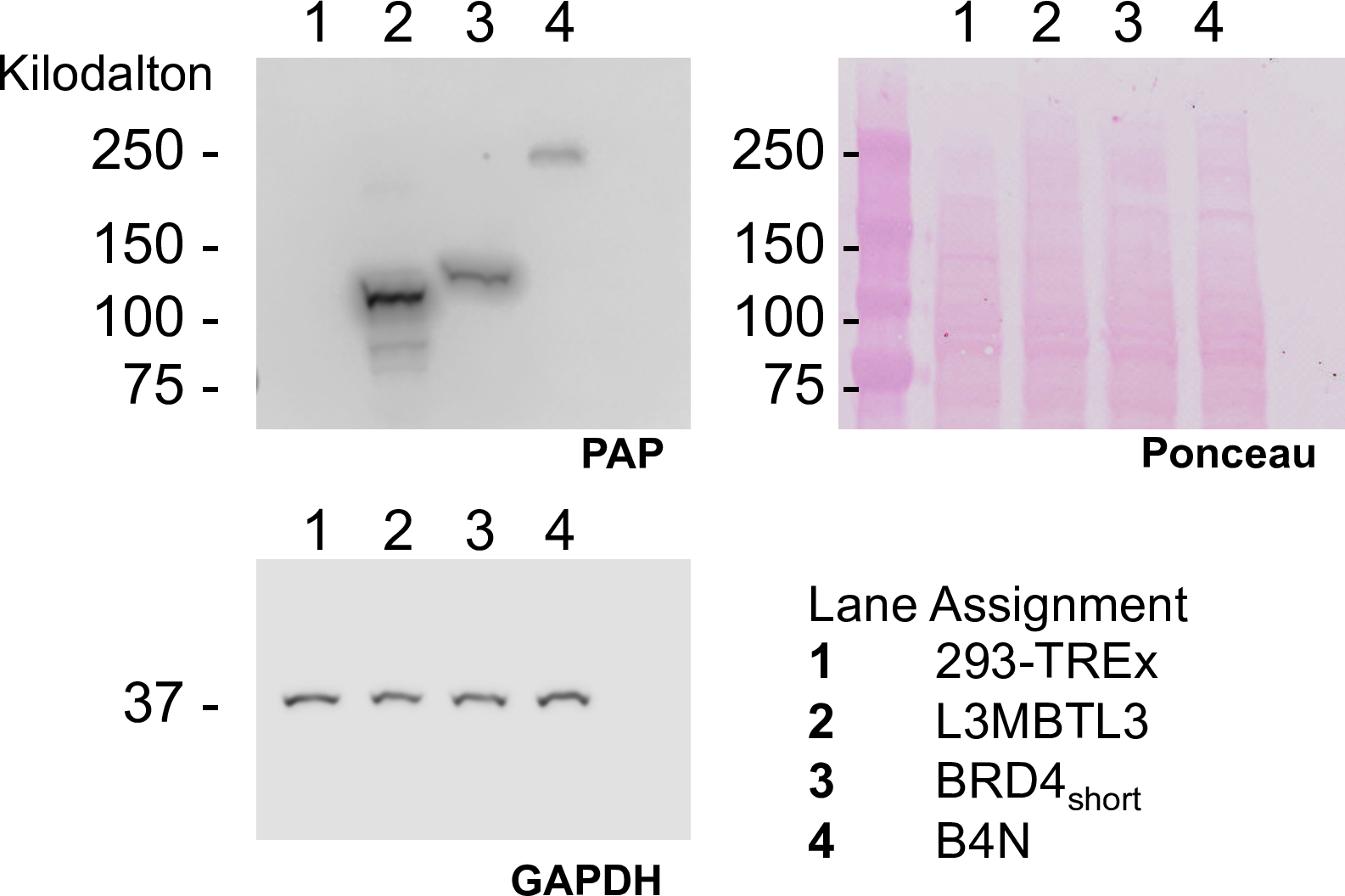

Supplement: S1 Fig — Western Blot analysis of protein extracts from tetracycline induced 293-TREx that are non-transfected (Lane 1), expressing BioTAP-L3MBTL3 (Lane 2; 100kd expected molecular weight), BioTAP-BRD4short (Lane 2; 100kd expected molecular weight) (Lane 3), and BioTAP-B4N (Lane 4; 220 kd expected molecular weight). PAP detects BioTAP-tagged baits. GAPDH and Ponceau staining reveal equal loading of extracts. (TIF) [file pone.0163820.s001.tif]

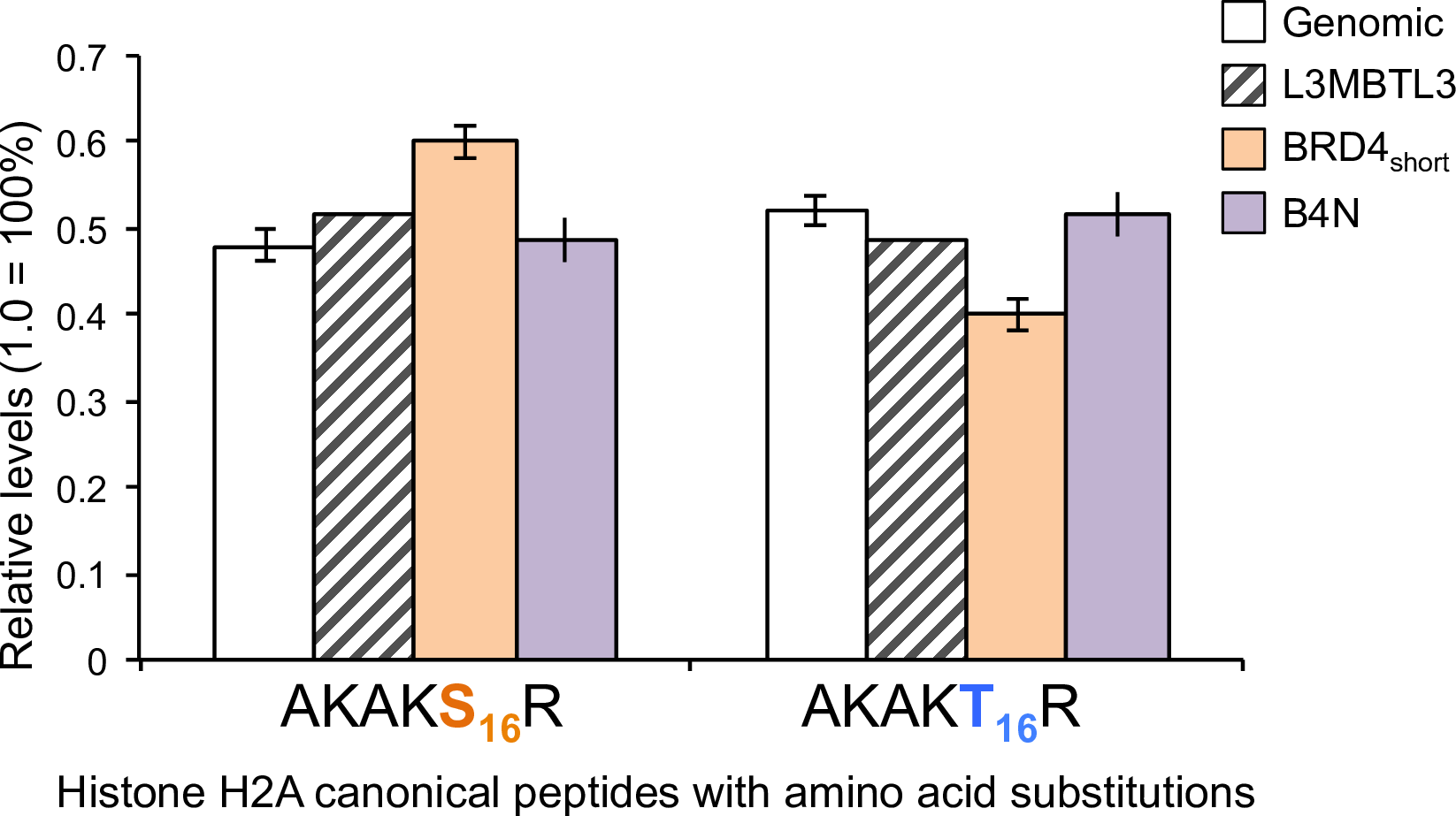

Supplement: S2 Fig — Recovery of H2A variant peptides across genomic and immunoprecipitated chromatin spanning endogenous substitutions at position 16 (serine compared to threonine). Error bar of genomic sample and BRD4short represents standard deviation of 4 replicates while error bar of B4N sample represents range of 2 replicates. (TIF) [file pone.0163820.s002.tif]

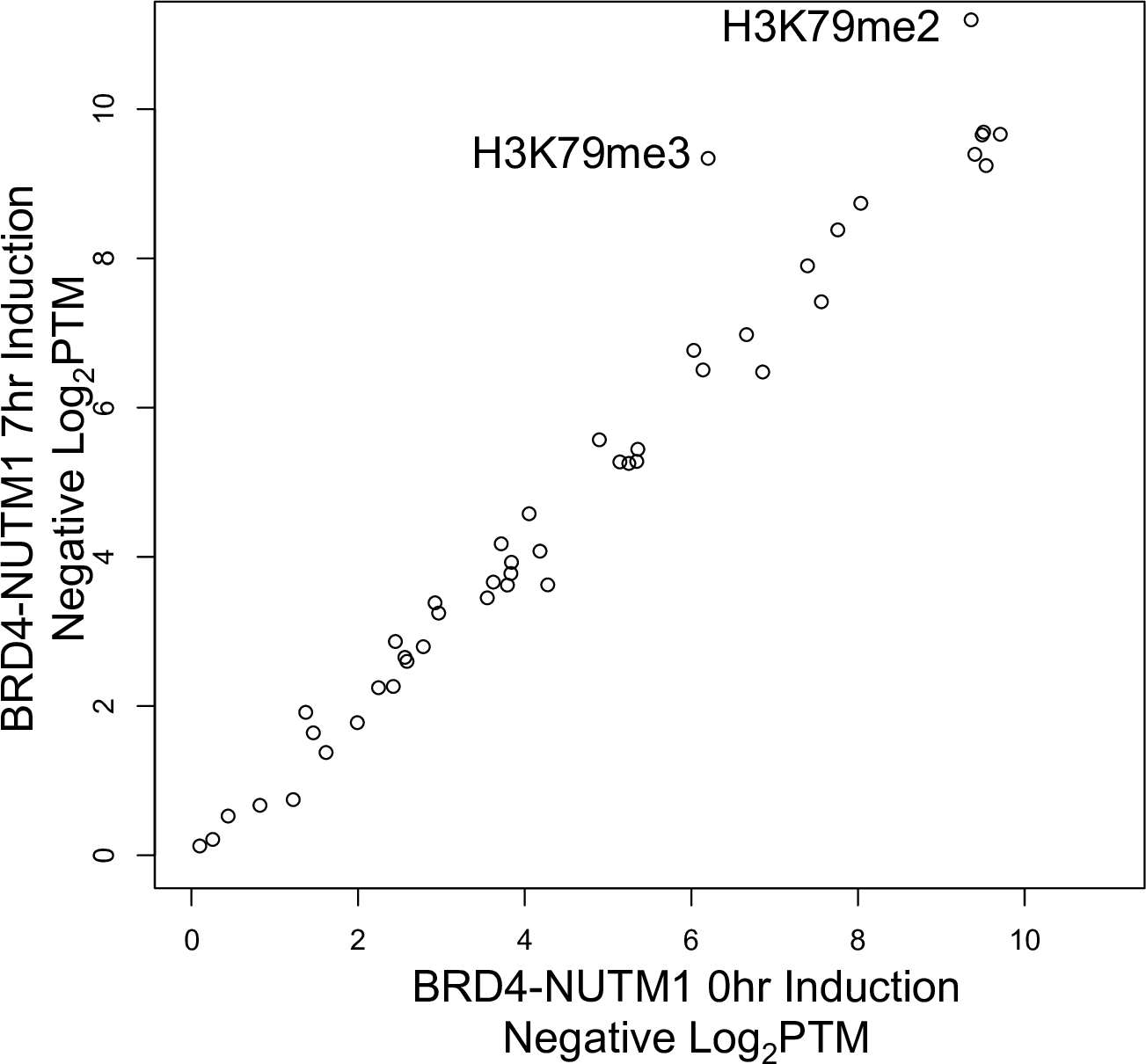

Supplement: S3 Fig — Comparison of global histone PTM levels in 293-TREx cells before and 7 hours after B4N induction. Each point in the plot represents a PTM. Relative PTM levels were determined as described in the report, where the area under the peak for each modified peptide was normalized to the sum of all the areas of the unmodified and modified forms of a given peptide backbone. The relative quantification was performed for both 0hr and 7hr induction, and the values per PTM at the two timepoints were log2 transformed and multiplied by -1. Note that, aside from H3K79 di- and tri-methylation, most PTMs on a genome wide scale do not significantly change upon B4N induction. (TIF) [file pone.0163820.s003.tif]

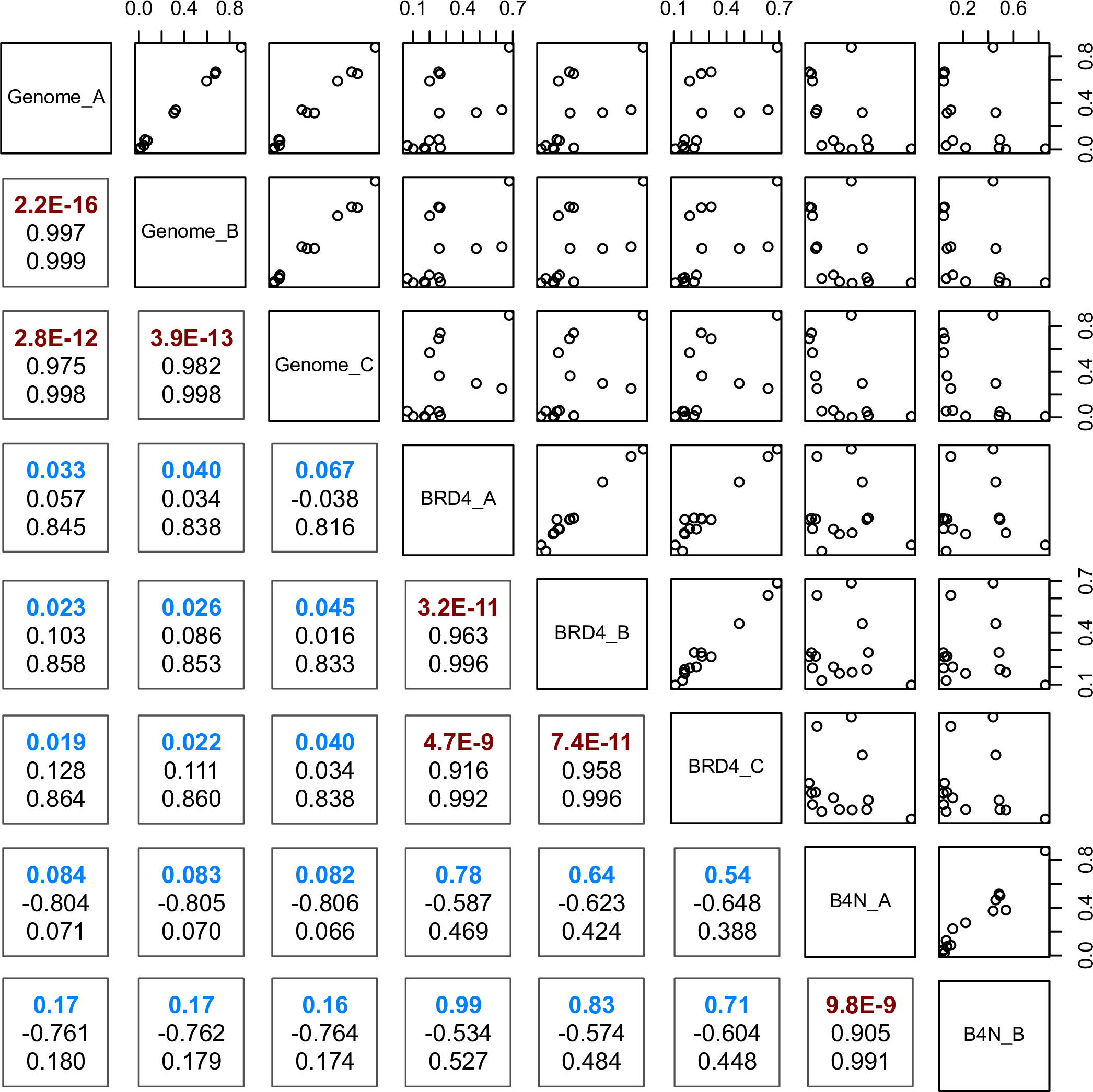

Supplement: S4 Fig — Pearson’s coefficient r was determined for the collective relative abundances of histone PTMs in pairwise comparisons between our samples (genomic input, BRD4short, and B4N). Above the diagonal are scatterplots of each comparison, with each point representing a histone PTM. For each comparison, we tested the likelihood that we would have observed the sample correlation if our null hypothesis of r being 0 were true. Thus low p-values suggest a high likelihood that there is a correlation. Statistical values are shown below the diagonal, where p-values are bolded and the lower and upper 95% confidence interval for Pearson’s coefficient are shown below. We find that within replicates, our MS-based PTM quants are tightly correlated. However, the B4N samples are not correlated (positively or negatively) with either input or BRD4short, suggesting that the B4N-associated PTM patterns as a whole are highly distinct from input and BRD4short. (TIF) [file pone.0163820.s004.tif]

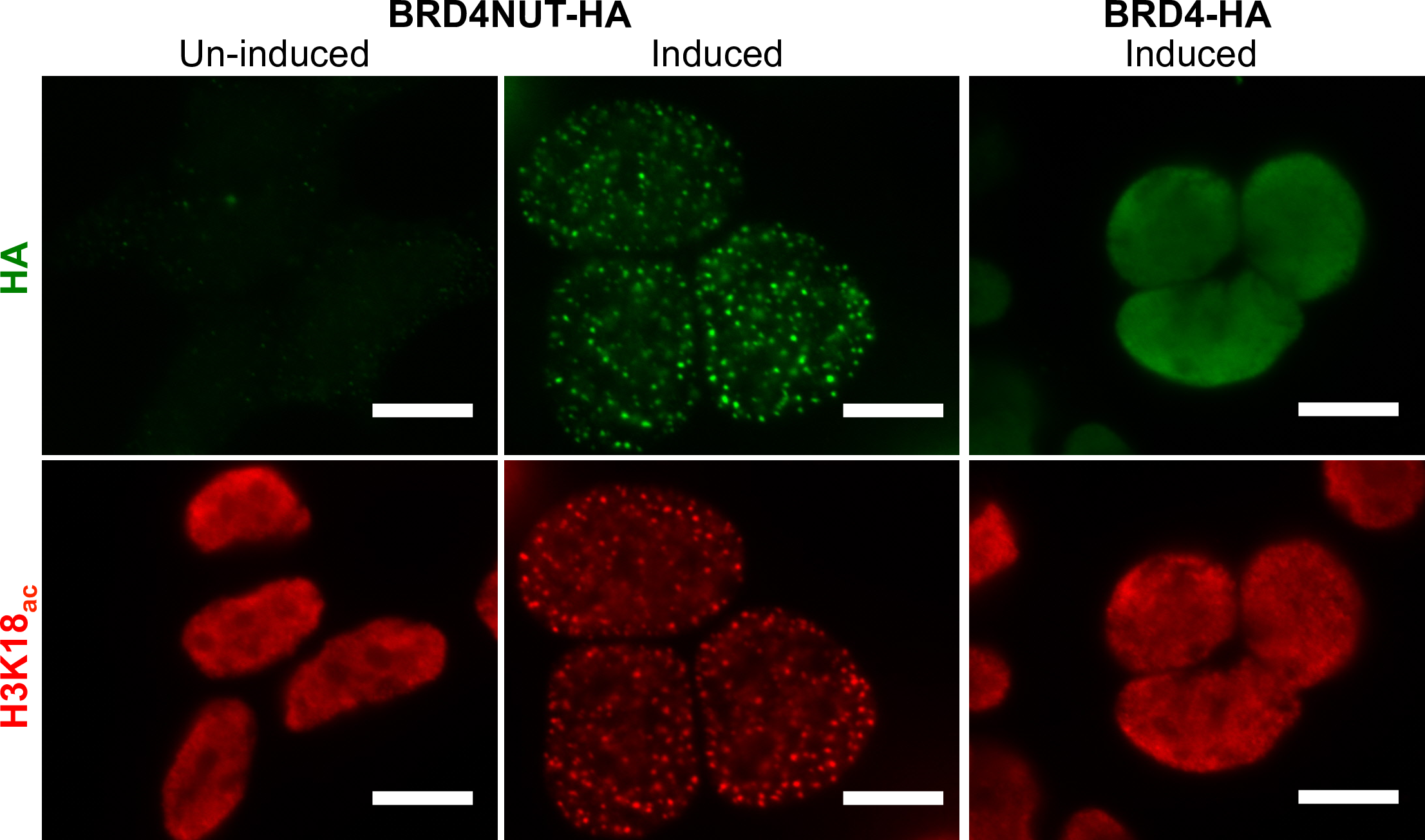

Supplement: S5 Fig — Images of 293-TREx cells expressing inducible HA-B4N and HA-BRD4short stained with anti-HA (green), which labels the bait protein, and anti-H3K18ac (red), acquired on non-confocal widefield fluorescence microscopy. Scale bar represents 10 micrometers. (TIF) [file pone.0163820.s005.tif]

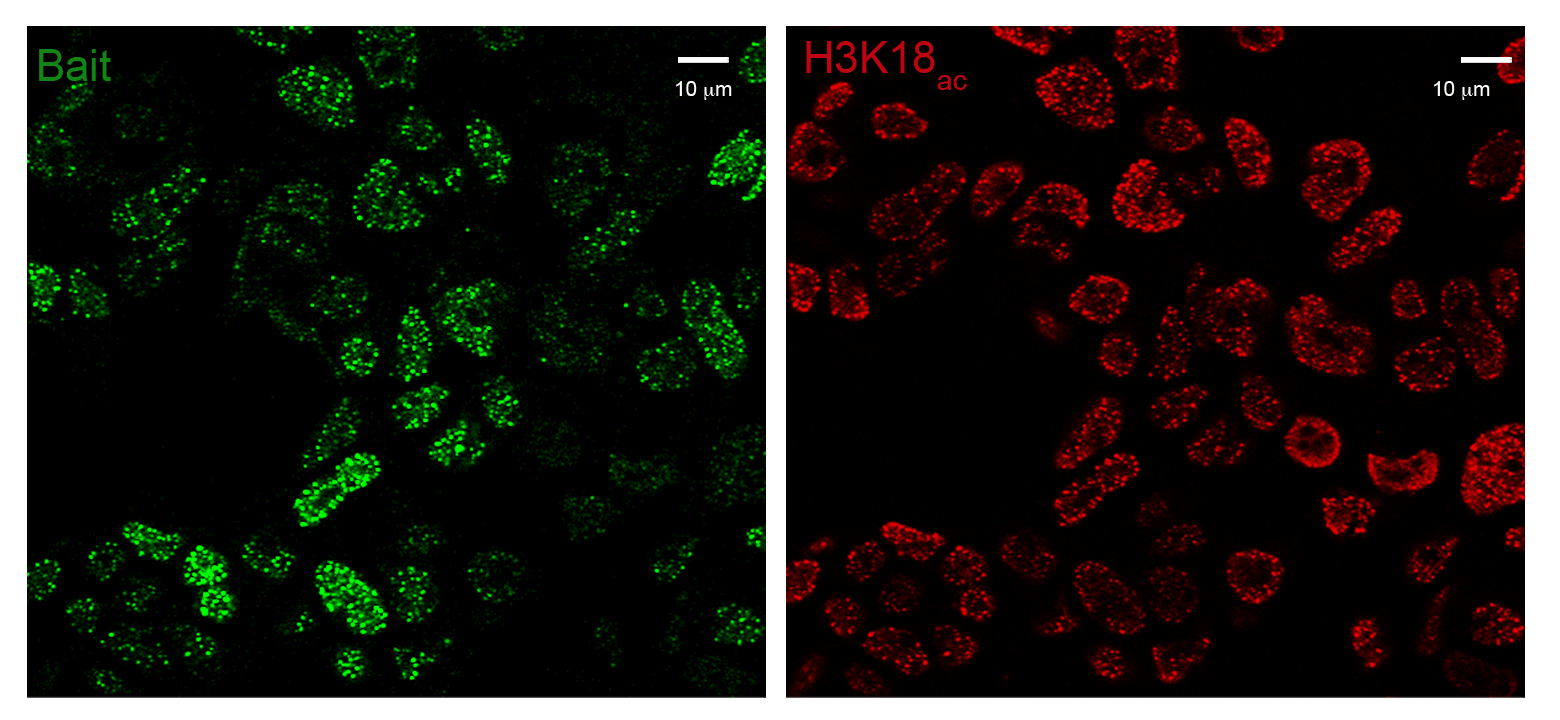

Supplement: S6 Fig — Same confocal image as Fig 3 with separate panels for the green (left, stained for BRD4-NUT bait) and red (right, stained for H3K18ac) channels for clarity. (TIF) [file pone.0163820.s006.tif]

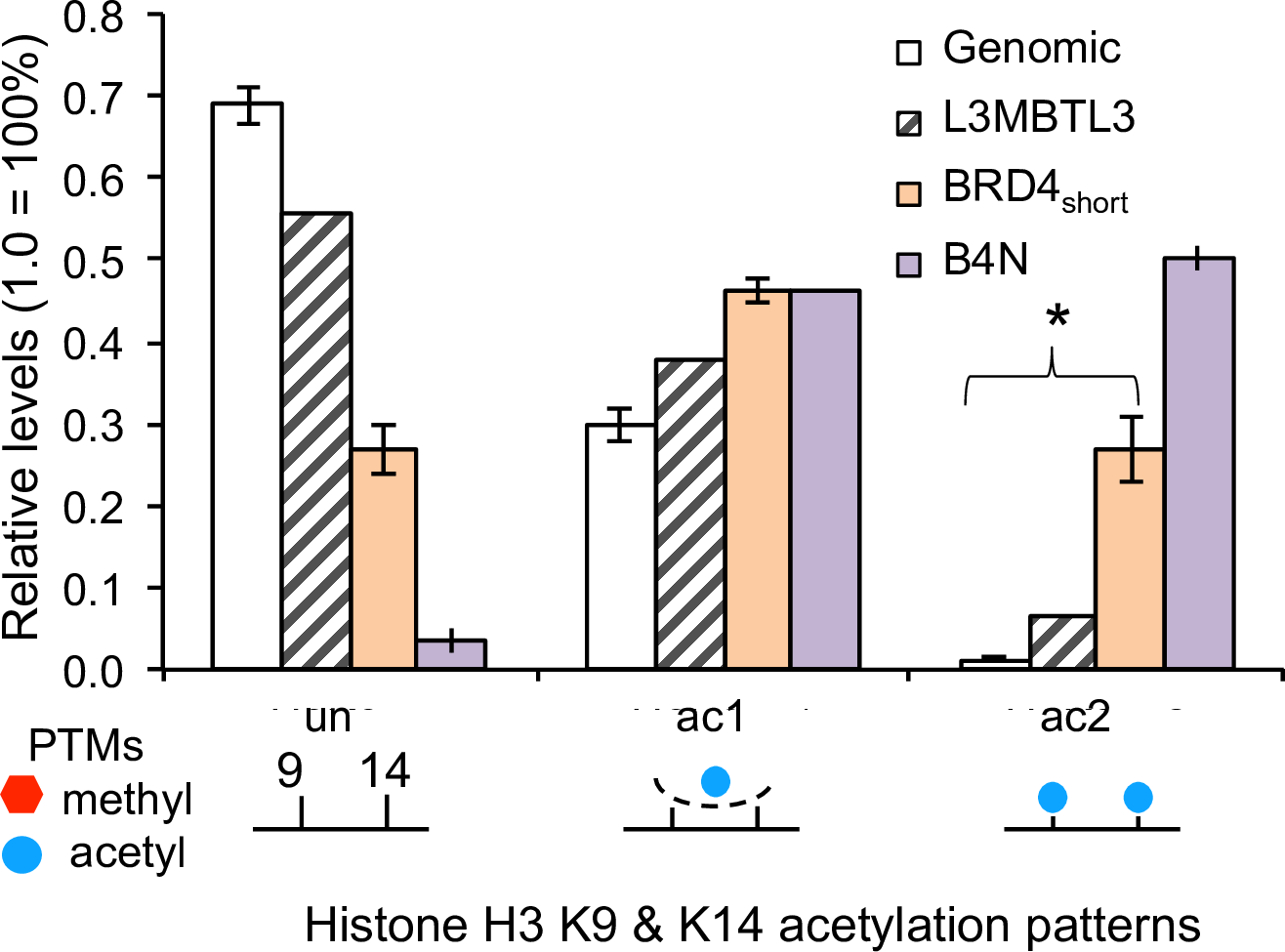

Supplement: S7 Fig — Relative levels of unmodified, mono-acetylation and di-acetylation of the H3 peptide spanning K9 and K14 in genomic and immunoprecipitated histones. Error bar of genomic and BRD4short sample represents standard deviation of 4 replicates. Error bar of B4N sample represents range of 2 replicates. Asterisk denote significant difference in H3K9K14ac2 levels between input and BRD4short (p < 0.05). (TIF) [file pone.0163820.s007.tif]

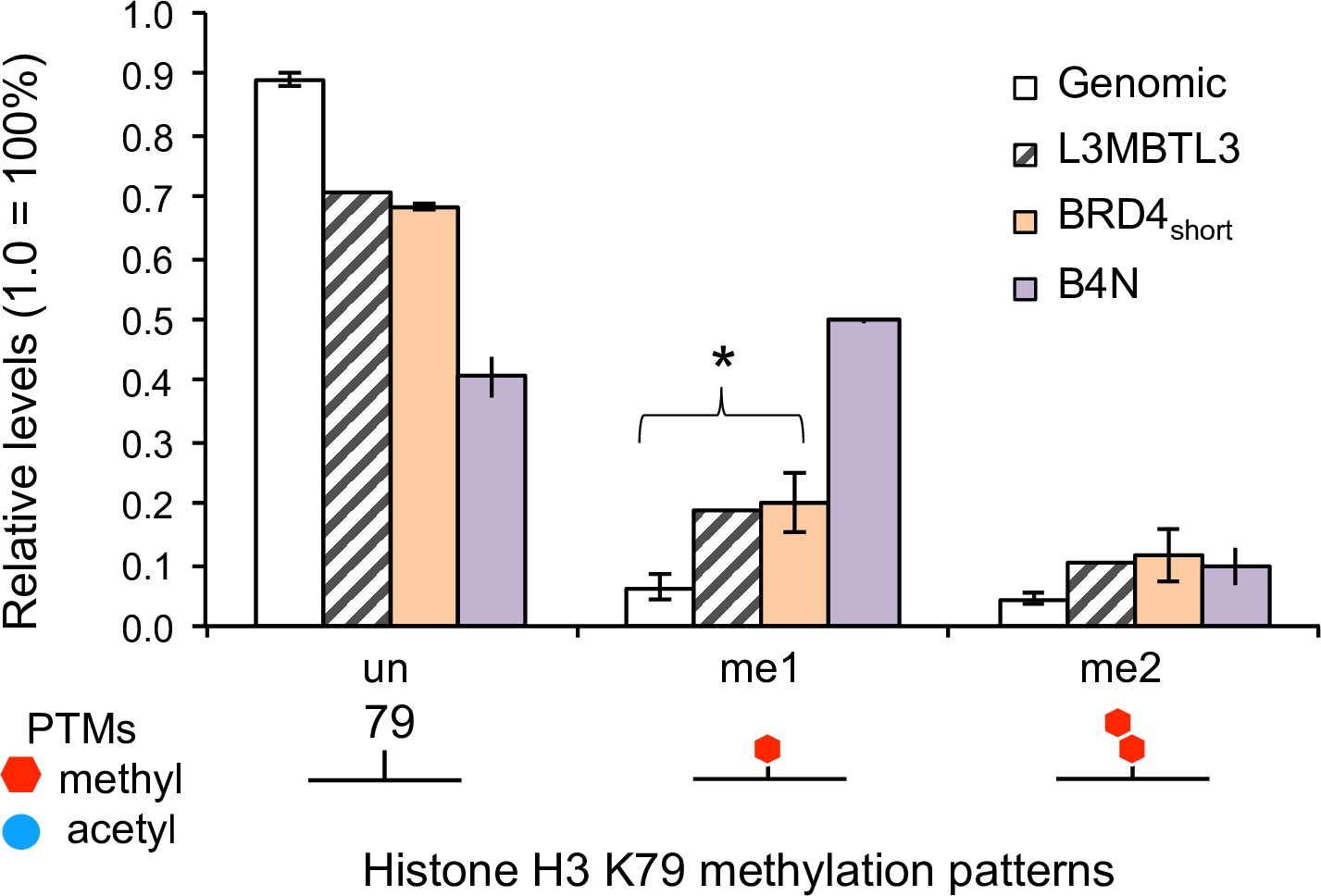

Supplement: S8 Fig — Relative levels of unmodified, mono-methylated and di-methylated H3K79 in genomic and immunoprecipitated histones. Error bar of genomic and BRD4short sample represents standard deviation of average of 3 replicates. Error bar of B4N sample represents range of 2 replicates. Asterisk denote significant difference in K79me1 levels between input and BRD4short (p < 0.05). (TIF) [file pone.0163820.s008.tif]

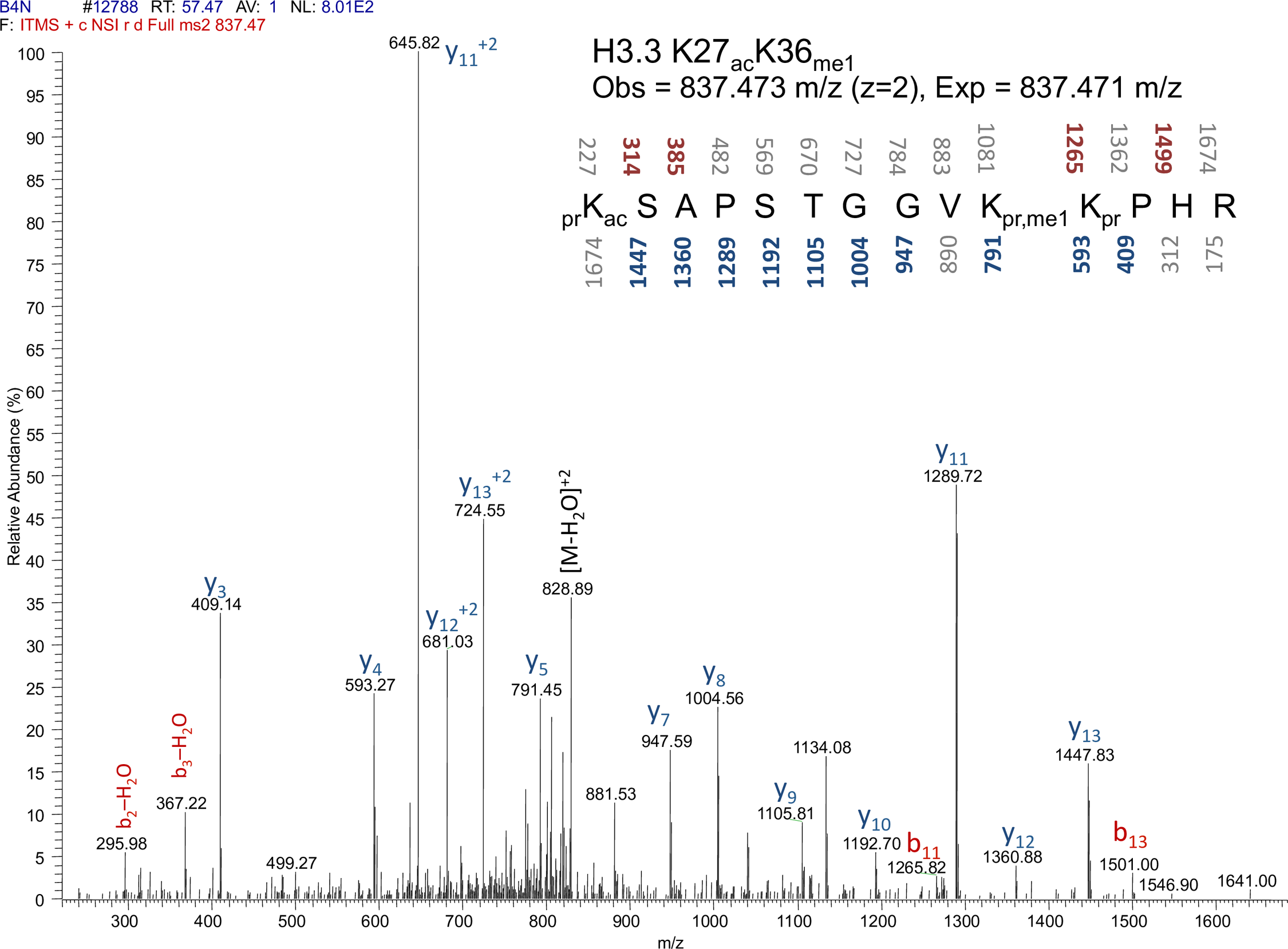

Supplement: S9 Fig — Annotated tandem mass spectrum of the H3.3 peptide containing K27 acetylation and K36 monomethylation. Numbers above and below peptide sequence correspond to nominal masses of b and y fragment ions. Bolded masses indicate those ions annotated from the spectrum. Pr = propone group from derivatization. (TIF) [file pone.0163820.s009.tif]

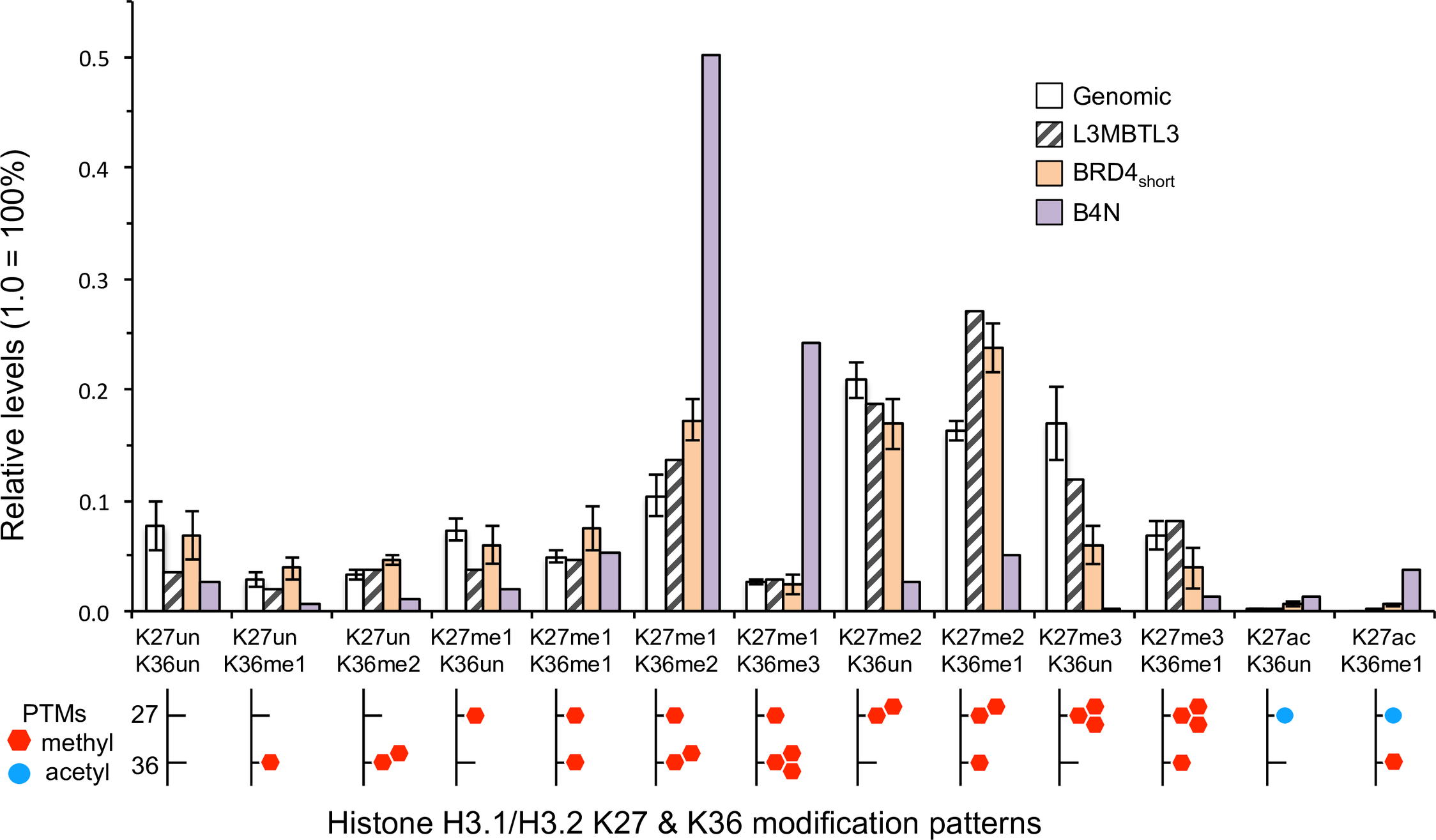

Supplement: S10 Fig — Relative levels of combinatorially modified forms of K27 and K36 methylation and acetylation. Error bar of genomic and BRD4short sample represents standard deviation of 4 replicates. Note the significant enrichment for K27 mono-methylation paired with K36 di- and tri-methylation by B4N. (TIF) [file pone.0163820.s010.tif]

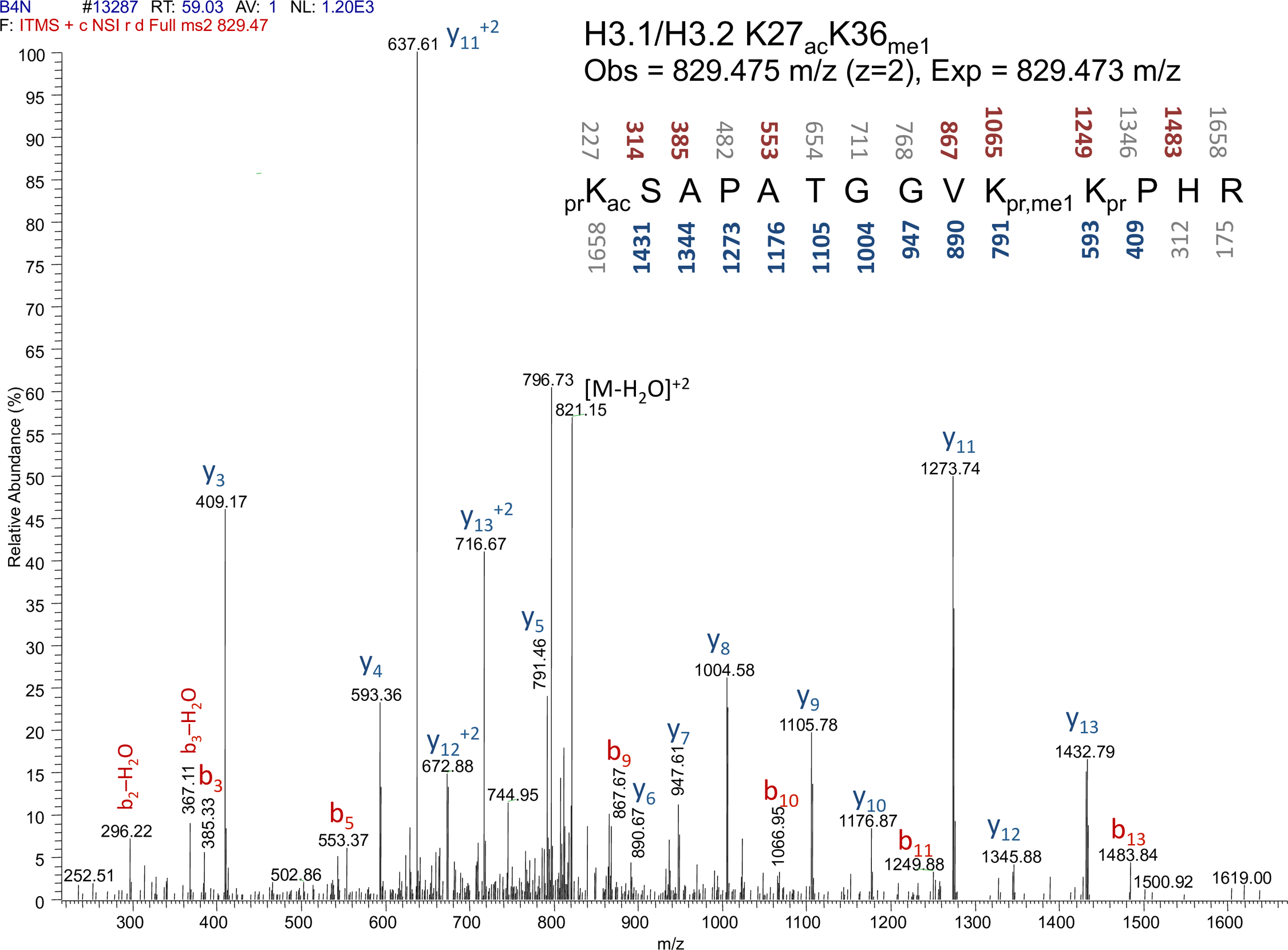

Supplement: S11 Fig — Annotated tandem mass spectrum of the canonical histone H3 peptide containing K27 acetylation and K36 monomethylation. Numbers above and below peptide sequence correspond to nominal masses of b and y fragment ions. Bolded masses indicate those ions annotated from the spectrum. Pr = propone group from derivatization. (TIF) [file pone.0163820.s011.tif]
